# Non-traditional cognitive brain network involvement in insulo-Sylvian gliomas: a case series study and clinical experience using Quicktome

**DOI:** 10.1186/s41016-023-00325-4

**Published:** 2023-05-26

**Authors:** Zhiqiang Wu, Guanjie Hu, Bowen Cao, Xingdong Liu, Zifeng Zhang, Nicholas B. Dadario, Qinyu Shi, Xiao Fan, Yao Tang, Zhangchun Cheng, Xiefeng Wang, Xia Zhang, Xiaorong Hu, Junxia Zhang, Yongping You

**Affiliations:** 1 Department of Neurosurgery, The First Affiliated Hospital of Nanjing Medical University, Nanjing, 210029 China; 2 Robert Wood Johnson Medical School, Rutgers University, Newark, NJ 08901 USA; 3 International Joint Research Center On Precision Brain Medicine, XD Group Hospital, Shaanxi Province, Xi’an, 710077 China; 4 Institute for Brain Tumors, Jiangsu Collaborative Innovation Center for Cancer Personalized Medicine, Nanjing Medical University, Nanjing, 211166 China

**Keywords:** Connectome, Brain network, Machine learning, Insulo-Sylvian gliomas, Cognitive function

## Abstract

**Background:**

Patients with insulo-Sylvian gliomas continue to present with severe morbidity in cognitive functions primarily due to neurosurgeons’ lack of familiarity with non-traditional brain networks. We sought to identify the frequency of invasion and proximity of gliomas to portions of these networks.

**Methods:**

We retrospectively analyzed data from 45 patients undergoing glioma surgery centered in the insular lobe. Tumors were categorized based on their proximity and invasiveness of non-traditional cognitive networks and traditionally eloquent structures. Diffusion tensor imaging tractography was completed by creating a personalized brain atlas using Quicktome to determine eloquent and non-eloquent networks in each patient. Additionally, we prospectively collected neuropsychological data on 7 patients to compare tumor-network involvement with change in cognition. Lastly, 2 prospective patients had their surgical plan influenced by network mapping determined by Quicktome.

**Results:**

Forty-four of 45 patients demonstrated tumor involvement (< 1 cm proximity or invasion) with components of non-traditional brain networks involved in cognition such as the salience network (SN, 60%) and the central executive network (CEN, 56%). Of the seven prospective patients, all had tumors involved with the SN, CEN (5/7, 71%), and language network (5/7, 71%). The mean scores of MMSE and MOCA before surgery were 18.71 ± 6.94 and 17.29 ± 6.26, respectively. The two cases who received preoperative planning with Quicktome had a postoperative performance that was anticipated.

**Conclusions:**

Non-traditional brain networks involved in cognition are encountered during surgical resection of insulo-Sylvian gliomas. Quicktome can improve the understanding of the presence of these networks and allow for more informed surgical decisions based on patient functional goals.

## Background

Performing intra-axial tumor surgery around the Sylvian fissure, insula, and opercular cortices has a significant risk of causing new and permanent neurological deficits because of the complex anatomical relationship of the insula with surrounding brain structures [1]. Although the regions and tracts associated with cognitive functions are attempted to be protected as much as possible, many patients still suffer from severe cognitive deficits after surgery. While it is generally wise to avoid cutting through white matter bundles, removing an intra-axial brain tumor always requires resecting some brain tissue, reflecting our constant need to make trade-offs in surgical choices. Given that neurocognitive decline occurs after brain surgery in many cases [2], even when there are no serious transgressions of white matter bundles, it is clear that additional information is necessary to define the anatomy at risk in these operations.

Connectomics provides a unified, network-based view dedicated to mapping and understanding the structural and functional topology of the human brain, revealing that the brain is a navigable tangle of integrated circuits [3]. However, the challenge has been to translate this revised cartography to the surgical sphere. Recent studies have clearly established the central role of large-scale brain networks, such as the central executive network (CEN) [4–6], default mode network (DMN) [7–10], salience network (SN) [5, 11–13], dorsal attention network (DAN) [4, 14, 15], and ventral attention network (VAN) [15], involving numerous aspects of human cognition. These networks include spatially distant, but highly synchronized brain regions and their connecting white matter fibers which dynamically work together across the human cortex. When damaged by brain tumors or inadvertently injured during surgery, damage to these networks can cause patients to experience cognitive morbidity after surgery [16].

Modern imaging and brain mapping technologies have assisted surgical procedures to achieve maximum tumor resection while preserving brain functions [17]. Unfortunately, progress has been held back by the substantial challenge of meaningfully mapping structural connections and understanding where gliomas have distorted and/or invaded these connections. The Quicktome™ (Quicktome, Sydney, Australia) is a Food and Drug Administration (FDA)-approved software which based on machine learning that creates a subject-specific version of the Human Connectome Project Multi-Model parcellation version 1 (HCP-MMP1) atlas [18]. This is performed by using reparcellation based on structural connectivity instead of anatomic-based methods, which is more accurate in patients with a distorted anatomy, as those with brain tumors [19, 20]. It requires two MRI sequences: a high-resolution diffusion tensor imaging (DTI) scan and T1-weighted contrast-enhanced anatomical scan. The platform allows to visualize large-scale brain networks, all the HCP Atlas parcellations segmented into the subject’s brain, and most tractography bundles.

Therefore, our primary aim was to utilize Quicktome to characterize a cohort of patients with gliomas around the insula and Sylvian fissure related to traditional and non-traditional brain networks, while we expect these cases will demonstrate that often these tumors are in areas well known to neurosurgeons to be “eloquent” structures, like the language system and sensorimotor cortices. Our specific goal was to demonstrate the frequency of invasion and/or proximity to portions of non-traditional, large-scale brain networks such as the CEN, DMN, SN, VAN, and DAN. Then, we retrieved a case series to evaluate the preoperative cognitive function status and whether it is consistent with the non-traditional brain networks involved. Finally, we reported our initial practice of using Quicktome as a pre-surgical tool to optimize the surgical approach in patients with insulo-Sylvian gliomas for preserving as much cognitive function as possible after surgery.

## Methods

### Patient selection and data collection

We retrospectively analyzed a series of adult patients undergoing surgery for newly diagnosed glioma at our center between 2017 and 2021 with WHO grade II to IV infiltrating gliomas centered in the insula, opercular cortices, or temporal stem. While many of these tumors extended beyond these regions, we included patients where the primary epicenter of the tumor was in the insulo-Sylvian regions. All of these patients received standard structural T1- and T2-weighted images and DTI for image guidance immediately before surgery. Then, we prospectively collected data on patients diagnosed with low-grade gliomas, glioblastomas, and recurrent gliomas in the same neurosurgical center from March to June 2022. Data collected included demographics, tumor location, primary symptoms, and functional independence (using the Barthel Index) before surgery. We also evaluated cognitive function using the Mini-Mental State Examination (MMSE) and the Montreal Cognitive Assessment (MoCA). These patients also received the same imaging protocol. Finally, we reviewed the surgical workflow of two patients undergoing supratentorial intra-axial tumor resection surgery, in which we used Quicktome to assist the decision-making of the surgical approach and evaluate network preservation post-operatively. The patients with glioma in the prospective study received the neurocognitive assessment of structured clinical interviews conducted by two experienced doctors to ensure the reliability of the results. This study was approved by the local Human Research Ethics Committee of the First Affiliated Hospital of Nanjing Medical University (No. 2021-SR-442).

### Diffusion tractography preprocessing steps

The parameters of the DTI scan were as follows: Siemens Skyra 3.0 MRI scanner, with 10 *b* = 0 baseline image and a *b* = 1000 shell with 64 direction acquisition, FOV = 224 mm × 224 mm, slice thickness 2 mm, 0-mm gap between slices with no overlap, full brain coverage, isotropic voxels, and square 112 × 112 matrix. The DTI was processed using the Quicktome Brain Mapping Platform (Omniscient Neurotechnology, Australia) [19], a cloud-based Health Insurance Portability and Accountability Act–compliant tool. The Quicktome employs standard processing steps in Python language [21], including the following steps: (1) the diffusion image is resliced to ensure isotropic voxels; (2) motion correction is performed through a rigid body alignment; (3) slices with excess movement (defined as DVARS > 2 sigma from the mean slice) are eliminated; (4) the T1 image is skull stripped through a convolutional neural net, and this is inverted and aligned to the DT image with a rigid alignment, which is then utilized as a mask to skull strip the DT; (5) gradient distortion correction is undergone using a diffeomorphic warping method to locally similarize the DT and T1 images; (6) eddy current correction is performed; (7) fiber response function is estimated, and the diffusion tensors are calculated using constrained spherical deconvolution; and (8) deterministic tractography is performed with random seeding, usually developing about 300,000 streamlines per brain.

### Creation of a personalized brain atlas using machine learning-based parcellation

The Quicktome algorithm creates a machine learning-based, subject-specific version of the HCP-MMP1 [18] atlas based on diffusion tractography structural connectivity, which has been described in detail in previous studies [22]. In short, it was created by training a machine learning model on 200 normal adult subjects by first processing T1 and DTI images as above. The HCP-MMP1 atlas in NIFTI MNI space is then warped onto each brain to assess structural connectivity between each parcellation pair of the atlas and a set of regions including eight subcortical structures per hemisphere and the brainstem based on streamlines terminating in each region. This step both allows the generation of feature vectors (379 × 379 structural connectivity-based adjacency matrix) and generates a centroid of the parcellation utilized to constrain the voxels studied for assignment to a given parcellation to a plausible area in the vicinity of its typical position. These feature vectors for each region were then used as a training set and the data were modeled through the XGBoost method.

Then, this model is applied to the new subject by first warping the HCP-MMP1 atlas to the new brain and collecting a set of feature vectors of the connectivity of each voxel. The feature vectors are then used to determine whether each voxel belongs to a parcellation or region or not and if so to assign the voxel to that parcellation. It creates a version of the HCP-MMP1 atlas with subcortical components, which is not dependent on brain shape or pathologic distortion and which is specific for this subject, but comparable between subjects.

### Definition of tumor boundaries

Tumor boundaries were defined through the preoperative postcontrast T1 and T2 images. We utilized definitions of tumor boundaries to align with specific surgical goals as opposed to the strict definition of all T2 changes in high-grade gliomas being defined as tumors [23, 24]. Thus, the boundaries of enhancing high-grade gliomas were defined solely as the contrast-enhancing portion of the tumor. The boundaries of low-grade gliomas were defined as the T2 hyperintense portion of the tumor. Of note, none of these patients received bevacizumab or other therapies which could affect the imaging behavior in a clinically significant way.

The rationale for limiting analysis of high-grade tumors to the contrast-enhancing portions is two-fold: First, the T2 changes in most insulo-Sylvian high-grade gliomas are quite extensive, which would make this study biased towards overestimating the frequency of involvement of networks like CEN, DAN, and SN within the surgical approach (they would be involved or near the T2 change in basically every case). Second, given the location of these tumors, it is unlikely that even advocates of supramaximal resectable surgery would recommend chasing T2 changes inside the corticospinal tract, arcuate fasciculus, and basal ganglia. Thus, given that most neurosurgeons operating on a high-grade glioma centered within the region would be to remove as much contrast-enhancing tumor as possible, the analysis should be performed in a way that aligned with the surgical goals of these cases. Thus, it would provide an accurate snapshot of the nature of non-traditional network involvement within a standard surgical neuro-oncology workflow.

### The determination of network involvement in glioma cases

The Quicktome defines networks as a set of cortical regions, using the machine learning-based atlas described above and the fiber bundles connecting the component regions. This structural connectivity-based approach to atlas leverages the insight that specific cortical regions have been reproducibly and robustly demonstrated to include strong fMRI blood oxygen level-dependent (BOLD) signal correlation with other regions in the brain, in patterns consistent across individuals. These correlation patterns form the basis of the concept of large-scale brain networks. For instance, regions of the brain which synchronize their activity because they serve a standard function. The cortical regions utilized in Quicktome to be the anatomic components of these networks are identified from published meta-analyses that mapped the specific large-scale brain networks to these specific regions [25–30]. We visually inspected the processed images to understand whether the boundary of the tumor was close to (defined by the tumor extending to within 1 cm of some portion of the tract or cortical region of a brain network template) or invaded various brain structures.

## Results

### Insulo-Sylvian gliomas usually involve both traditional and non-traditional brain networks

A total of 45 patients were included in the retrospective analyses of this study. The median age at diagnosis was 47 ± 15 years, with 22 being male. The demographic data is outlined in Table 1. Twenty-six tumors were located in the left hemisphere, and 19 were in the right hemisphere. All tumors were classified by WHO grade, in which 10 were grade II gliomas, 10 were grade III gliomas, and 25 grade IV gliomas. While most of these tumors showed some extension into the insula and many were multi-lobular or large tumors, only 6 of these patients depicted a tumor primarily focused in the insula. In contrast, 13 had tumors mostly focused in the frontal lobe and frontal opercula, 22 had primarily temporal tumors, and four tumors were mainly focused within the parietal opercula and inferior parietal lobule.

**Table 1:** Demographic characteristics of patients with insulo-Sylvian glioma

**Variable (*n* = 45)**	
Age (mean, SD, in years)	47 (15)
*Gender*	
Female (*n* (%))	22 (49%)
*Ipsilesional side*	
Left (*n* (%))	26 (58%)
*WHO grade (n (%))*	
Grade II gliomas	10 (22%)
Grade III gliomas	10 (22%)
Grade IV gliomas	25 (56%)
*Location of the tumor (n (%))*	
Insula	6 (13%)
Frontal predominant	13 (29%)
Temporal predominant	22 (49%)
Parietal predominant	4 (9%)

We observed that if we limited our analysis to whether an insulo-Sylvian glioma was close to or invading a structure classically viewed as eloquent by neurosurgeons (particularly the corticospinal tract, the motor cortex, the language regions or tracts, the optic radiations, or the basal ganglia), around 77% of patients (35/45) demonstrated the involvement of a traditional eloquent structure. Conversely 44/45 (98%) patients showed involvement (proximity or invasion) of a non-traditionally eloquent brain network, such as the CEN, SN, DMN, DAN, or VAN. Ultimately, only one patient in this series did not demonstrate a surgically relevant risk to some structure, network, or tract associated with a known neurological or cognitive function. An overall summary of the patterns of a brain network involved with these insulo-Sylvian tumors is displayed in Table 2. Notably, the SN and associated frontal aslant tract (FAT) were frequently involved, being nearby or invaded in 60% of these cases, along with other more laterally positioned networks, CEN (56% of cases) and VAN (63% of right-sided cases as it is a right-lateralized network). The more medial positioned DAN was affected slightly less often (47% of cases), and the DMN was relatively uncommon (18% of cases) with the involvement being most often due to proximity to the parietal component of the DMN having the medial frontal component invaded by tumor extension in one case.

**Table 2:** Overall summary of the patterns of network involvement with these insulo-Sylvian tumors

		**Uninvolved**	**Closer than 1 cm**	**Structure invaded**
Cognitive networks	Salience	18 (40.00%)	9 (20.00%)	18 (40.00%)
	CEN	20 (44.44%)	8 (17.78%)	17 (37.78%)
	DMN	37 (82.22%)	1 (2.22%)	7 (15.56%)
	DAN	24 (53.33%)	9 (20.00%)	12 (26.67%)
	VAN	32 (71.11%)	4 (8.89%)	9 (20.00%)
Traditional eloquent structures	Optic radiations	16 (35.56%)	22 (48.89%)	7 (15.56%)
	Language	19 (42.22%)	7 (15.56%)	19 (42.22%)
	Motor cortex	20 (44.44%)	10 (22.22%)	15 (33.33%)
	CST	10 (22.22%)	24 (53.33%)	11 (24.44%)
	Basal ganglia	19 (42.22%)	20 (44.44%)	6 (13.33%)
White matter tracts	FAT	18 (40.00%)	12 (26.67%)	15 (33.33%)
	IFOF	14 (31.11%)	17 (37.78%)	14 (31.11%)
Analysis limited to relevant hemisphere	Language	0 (0%)	7 (26.92%)	19 (73.08%)
	VAN	7 (36.84%)	4 (21.05%)	8 (42.11%)

### Cognitive decline is typical in the insulo-Sylvian glioma patients

During the second part of the study, we prospectively analyzed a total of seven glioma patients with insular infiltration in the same hospital. The median age was 55 ± 8 years (ranging from 45 to 68), with women being 4 of these patients. The Quicktome demonstrated that the SN and the associated FAT were involved in all cases, while the traditional networks, such as sensorimotor network (6/7, 86%) and language system (5/7, 71%), were involved in most cases. In addition, the CEN (5/7, 71%) and the language system (5/7, 71%) were also the major involved brain networks in these seven patients. The clinical performance and cognitive evaluation of these patients were consistent with the indications from Quicktome. Fifty-seven percent (4/7) of the patients complained of memory decline and communication disorders. The pre-surgical mean MMSE score was 18.71 ± 6.94 (range from 5 to 25), and the mean score of MOCA was 17.29 ± 6.26 (range from 6 to 25), indicating that all the patients were in a declined cognitive function status. The general functional independence before surgery was in accordance with the severity of cognitive decline. Although the average score of the Barthel Index was 84.29 ± 23.71, the patient with the most severe score (Barthel Index = 45) was partially dependent and they also had the most severely affected cognitive function. The demographic characteristics, tumor location, traditional and non-traditional brain network involvement, and the cognitive and functional independence status are demonstrated in Table 3.

**Table 3:** Demographics and clinical information of 7 independent patients with insulo-Sylvian glioma

ID	Age	Sex	Tumor location	Traditional network involved	Non-traditional involved	Symptoms	MMSE	MOCA	Barthel Index
1	45	M	L temporal/insula	Sensorimotor	SN	Right figures numbness	25	25	100
2	54	F	R temporal/insula/frontal	Sensorimotor	SN/VAN/CEN/DMN	Seizures	23	22	100
3	59	F	L temporal/insula/parietal	Sensorimotor/language	SN/CEN/DMN	Memory decline and speech disorders	18	17	95
4	58	F	L temporal/insula/frontal	Language	SN/CEN/DEN	Memory decline and dizziness	23	18	100
5	68	M	L temporal/insula	Sensorimotor/language	SN/CEN/OR	Memory decline, right-side vision decline, dizziness, and headache	15	13	55
6	54	F	L temporal/insula	Sensorimotor/language	SN/CEN/DMN	Headache and cognitive disorders	5	6	45
7	56	M	L temporal/insula	Sensorimotor/language	SN	Transient syncope	22	20	95

*F* female, *M* male, *Oligo* oligodendroglioma, *GBM* glioblastoma, *R* right, *L* left, *VAN* ventral attention network, *SN* salience network, *CEN* central executive network, *DMN* default mode network, *OR* optic radiations

### Quicktome can optimize the surgical approach to preserve cognitive function

Consequently, we reported two cases in which we used Quicktome to assist surgical decision-making and predict the preservation of cognitive function after surgery. Of note, the final surgical decisions were not made by Quicktome alone but were recommended based on our usual clinical practice. During the surgery, neuronavigation (BrainLab) was set up after the patient’s head was fixed with the head holder. The Quicktome was integrated into this neuronavigation platform.

**Fig. 1:**
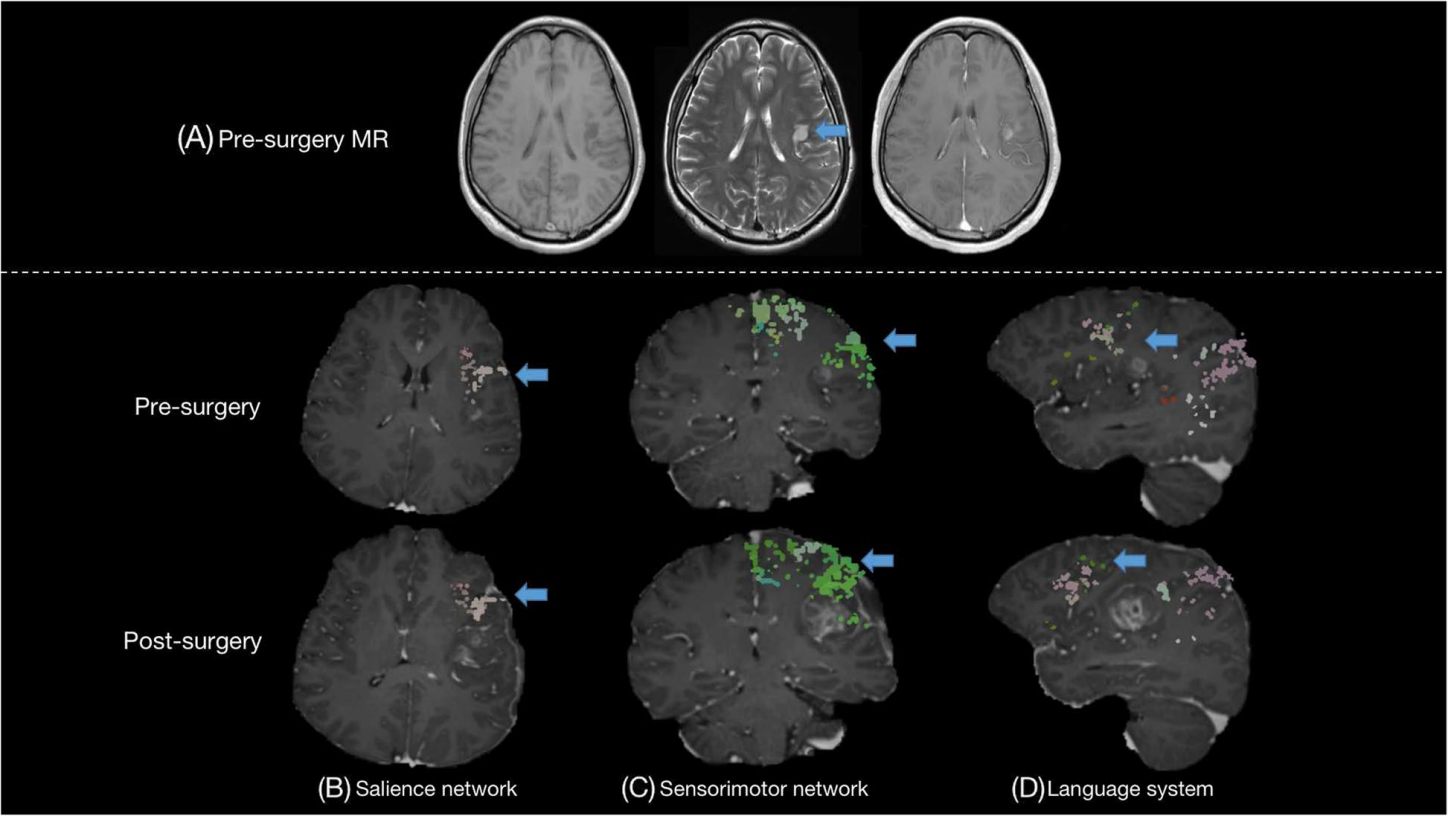
MRI images and the networks showed by the Quicktome in patient no. 1, who had the glioma resection in the left temporal and insula lobe. **A** T1, T2, and postcontrast T1 images before surgery; the Quicktome software demonstrates part of the **B** salience network, **C** sensorimotor network, and **D** language system, where were close to the tumor before surgery and the resection location after surgery

Patient no. 1 was a 45-year-old, right-handed male, who felt numbness in his right fingers for a month prior to admission. Preoperative MRI depicted a left insula glioma with high malignant potential (Fig. 1A). Considering that this small-size tumor did not involve the traditional speech and motor function networks, our initial surgical goal was a maximal tumor resection through the shortest transcortical distance, which would go through the precentral gyrus. However, Quicktome noted that the initial trans-cortical approach would damage the sensorimotor area and the SN (Fig. 1B, C, pre-surgery). Therefore, we optimized the surgical approach to remove the tumor through the postcentral gyrus and resect the anterior border of the tumor, which was very close to the precentral gyrus, to protect the sensorimotor area and the SN. We also avoided the superior temporal gyrus and Wernicke’s area during the excision of the posterior border of the tumor to avoid damaging the speech network (Fig. 1D, pre-surgery). The postoperative MRI showed that the tumor was completely resected and Quicktome also revealed complete preservation of the SN, sensorimotor network, and language system (see Fig. 1B–D, post-surgery). The patients retained normal cognitive function after the 3-month follow-up (MMSE score before vs after surgery: 25 vs 30, MoCA score before vs after surgery: 25 vs 26).

**Fig. 2:**
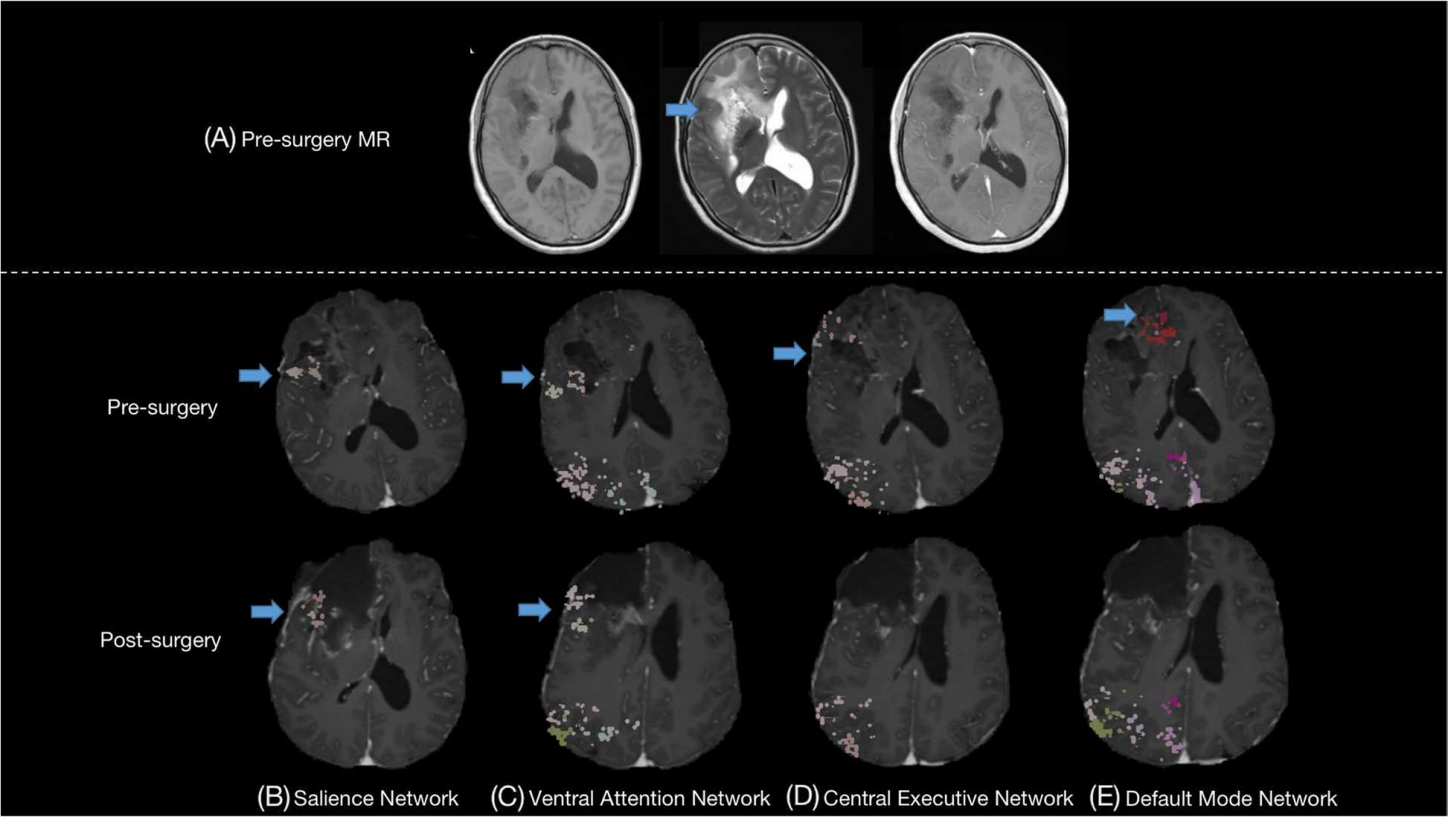
MRI images and the networks showed by the Quicktome in patient no. 2, who had the glioma resection in the right frontal, temporal, insula lobe. **A** T1, T2, and postcontrast T1 images before surgery; the Quicktome software demonstrates part of the **B** salience network, **C** ventral attention network, **D** central executive network, and **E** default mode network, where were inside or close to the tumor before surgery and the resection location after surgery

Patient no. 2 was a 54-year-old, right-handed female, who had been having absence seizures for 5 years and experiencing grand mal epileptic seizures every few months for 2 years without medication use. The frequency of her seizures increased the week prior to admission. Preoperative MRI showed a large, right-sided tumor invasion into the frontal, temporal, and insular lobes (Fig. 2A). The Quicktome noted that the SN and VAN were adjacent to the posterior border of the tumor in the middle of the inferior frontal gyrus, while the CEN and DMN were involved inside of the tumor (Fig. 2B–E, pre-surgery). Due to the extensive tumor infiltration, we chose the trans-frontal and temporal approach to achieve a gross total resection of the tumor, while avoiding damage to the SN and VAN. While the intraoperative fast-pathology revealed a high grade of malignant glioma, the resection was along the tumor border under the guide of intraoperative neuronavigation and ultrasound to reserve those critical networks and tracts related to cognitive function, whereas a supramaximal resection in this region was initially considered. The patient presented with a transient mutism immediately after waking from surgery but recovered to normal speech function two days after surgery. The postoperative MRI revealed preservation of the SN and VAN (Fig. 2B, C, post-surgery), which represented our effort to protect these networks with favorable cognitive functions. Although the DMN and CEN were inevitably damaged during the surgery (Fig. 2D, E, post-surgery), the patient still maintained the same cognitive function evaluated by the post-surgical neurological test at the 3-month follow-up (MMSE score before vs 3 months post-surgery: 23 vs 24, MoCA score before vs after surgery: 22 vs 22).

## Discussion

In this study, we found that greater than 98% of these patients had tumors that had sub-centimeter distance to the cortical or subcortical components of a large-scale brain network or major white matter pathway (or it invaded these structures). Most of these patients had multiple structures at risk from their tumors, highlighting the challenges posed by these complex tumors and suggesting that these cases seldom are no risk, even when in the right hemisphere. In the prospective case series study, cognitive function declined in all the cases, which happened to coincide with the non-traditional brain networks that were involved with the tumors. Based on an increased understanding of these essential non-traditional networks, we used Quicktome in the insular glioma resection surgery in two cases and explored its potential to provide support in pre-surgical planning and understand the complex functional connectomic networks and tracts of insular and peri-Sylvian gliomas. The following vital lessons and limitations derived from this study and the initial experience using Quicktome as a surgical assistant tool are discussed below.

### Connectome-based neurosurgery

A substantial amount of literature has been dedicated to refining techniques for reducing damage to traditional eloquent areas for mitigating post-surgical deficit risk. Recent efforts have focused on functional preservation techniques in less well-known anatomy, including preservation of the inferior frontal-occipital fasciculus (IFOF) [31] and FAT [32, 33]. Such outcomes provide evidence for patient-to-patient variability of the brain network architecture and speak to the need for a more refined understanding of brain connectivity. There are substantial advantages to reconsidering the deep white matter anatomy in terms of large-scale brain networks. Notably, many sizeable white matter bundles are in fact numerous bundles connecting different areas, not all of which are necessary for human cognitive function. This is particularly true for the superior longitudinal fasciculus (SLF)-arcuate fasciculus (AF) complex, a highly complex network of connections of varying importance. Since some parts of these bundles and their branches are more important than others, and we need to resect some areas to remove the glioma, network-based approaches provide the potential benefit for better defining which white fibers are more important than others. Ultimately, all brain surgery involves accurately calculating the risks against the rewards of our actions.

### Non-traditional brain networks related to cognitive function

Disturbance of motor, sensory, and language functions could significantly affect the quality of life for brain-damaged patients. However, the survivorship phase is often etched with disruptive cognitive functional impairments; 30–50% of survivors encounter significant cognitive and functional impairment after treatment, being one of the most concerning outcomes for patients [34]. Cognitive changes threaten the ability of glioma survivors to undergo complex daily life activities and limit their ability to return to work and perform meaningful and important roles [35]. However, it is challenging to investigate the effects of brain tumor surgery on cognitive functions such as memory, attention, and executive functions compared to motor and verbal functions, and brain regions that remain vulnerable to cognitive impairments have not been sufficiently investigated. In this study, we employed Quicktome to evaluate whether the tumor located at insulo-Sylvian regions had an impact on cognitive function and explored the non-traditional brain networks compared with traditional ones. In this retrospective study, 98% of patients demonstrated tumor involvement in the cortical or subcortical components of a non-traditional brain network or major white matter pathway involved in cognition. The most common non-traditional cognitive networks included the SN (60%), followed by the CEN (56%). Consistent with the above results, the prospective case series study revealed that the SN was involved in all seven patients and their cognitive function had also declined to different levels assessed using cognitive tests.

**Fig. 3:**
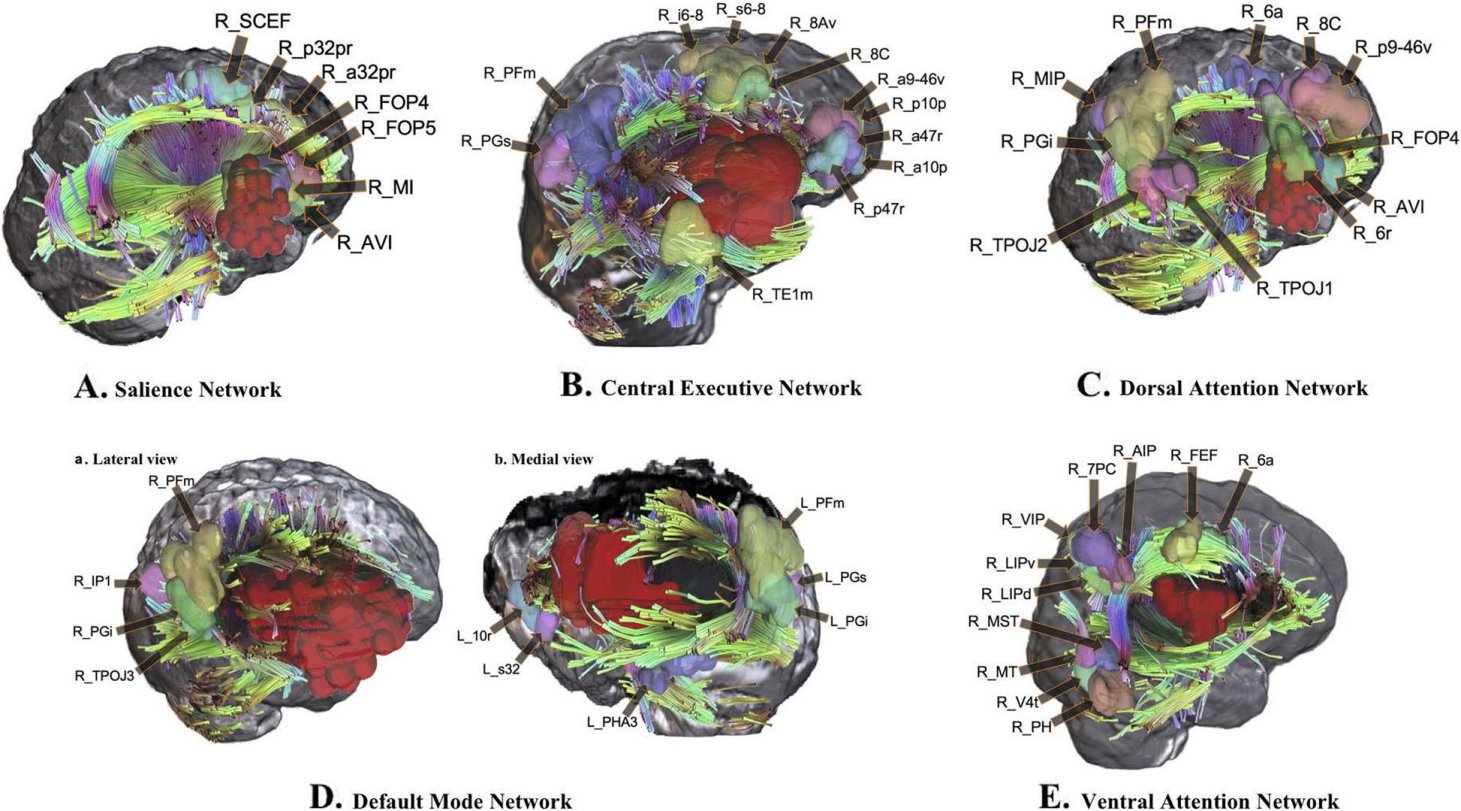
The typical examples to demonstrate the networks invaded by tumor. Tumor is represented in red, while the HCP parcels comprising the network have been labeled

The SN is integral for sensorimotor processing, general cognition, and coordination between emotion, pain, and physical action [36]. As the mind’s moderator, the SN constantly monitors the external environment and decides how other brain networks react to new information and stimuli. It also plays an essential role in switching between the internal and external processing [37] of the two main-control brain networks: the DMN and CEN [38]. The main functional areas of the SN are located in the anterior cingulate, the anterior insula [39, 40], and the presupplementary motor areas [12]. The SN also includes nodes inside the amygdala, hypothalamus, ventral striatum, thalamus, and specific brainstem nuclei [39], anterior cingulate cortex, medial temporal network, parahippocampal gyrus, olfactory lobe, and the ventral tegmental area (e.g., tumor invading the SN in Fig. 3A). The CEN is a superordinate control network [41] and uses input from other networks for task selection and executive function. By integrating with the other brain networks, the CEN processes various information, such as flexibility, working memory, initiation, and inhibition, all of which had previously been thought to be separate processes. Since its initial discovery within the anterior frontal lobe [42], the central executive network has been found to be functionally connected to the anterior cingulate cortex, the inferior parietal lobe [43], and the posterior most portions of the middle and inferior temporal gyri [44, 45](e.g., tumor invading the CEN in Fig. 3B).

The DMN, DAN, and VAN were also involved as the non-traditionally eloquent brain networks in the retrospective patient cohort. The DMN serves a primary role in the passive mind [46], like internal thought or contemplation. However, it is also active during some goal-oriented tasks. The DMN is typically described as consisting of the anterior and posterior cingulate cortices and the lateral parietal lobe bilaterally (e.g., tumor invading the DMN in Fig. 3D). The DAN is an essential mediator of goal-directed attentional processing [26], and it has many ways of contributing to intellectual capabilities. As a bilateral network, the DAN demonstrates robust connectivity between areas in the lateral occipital lobe, the pre-central sulcus, the dorsal-most portion of the superior frontal sulcus considered to be the frontal eye fields, the ventral premotor cortex, superior parietal lobule, intraparietal sulcus, and motion-sensitive middle temporal area [43] (e.g., tumor invading the DAN in Fig. 3C). The VAN is one of the two-network models of cortical attention [47, 48], which is involved in reorienting attention whenever a new, unexpected stimulus, like shock, frightening events, or “oddball” occurrences, is detected within the environment [48]. Multiple cortical areas, including the middle and inferior frontal gyri, anterior insula, inferior parietal lobule, and temporo-parietal junction have been linked in this processing [27] (e.g., tumor invading the VAN in Fig. 3E).

### Visualize non-traditional brain networks to preserve cognitive function

Barriers to translate the renewed understanding of the brain network architecture to clinical practice prevents surgeons from accessing valuable insight which has the potential to prevent the severe postoperative morbidity in higher-order functions seen in glioma patients [49–51]. The Quicktome shows its potential to provide neurosurgeons with the following information: (1) selection of an optimal trajectory to the tumor and awareness of the adjacent functional areas that need to be preserved and (2) assessment of the adjacent areas that may limit a supramaximal resection to preserve the cognitive functions.

Neurosurgeons usually select the shortest transcortical distance as the surgical approach to avoid traditional functional areas. The goal of maximal tumor resection should be balanced with potential neurological deficits that may result. To optimize the surgical approach and minimize the damage of surgery, our initial experience with Quicktome reveals its ability to visualize the essential brain networks and tracts to assist in surgical planning and decision-making, which allow neurosurgeons to provide more detailed explanations of the benefits and risks of the surgery to the patient, especially if the aggressive surgical resections are to be offered. For example, in the first case above, Quicktome indicated the possible damage to the sensorimotor area and the SN if we followed the initial surgical plan through the anterior central gyrus, so we changed to remove the tumor via the posterior central gyrus, to protect the motor and cognitive function. It is noticed that the functional areas of traditional and non-traditional neural networks were protected as much as possible after surgery. Indeed, the patient retains good motor and cognitive function after surgery, which confirmed these preserved tracts.

### Limitations and future directions

The specific goal of this study was to estimate how often a surgeon performing surgery for infiltrating gliomas around the Sylvian fissure could reasonably expect that a network not normally addressed at the surgery would be inside of the tumor, and this ultimately suggested that this was the anticipated nature of almost all of these tumors. This study was not intended to be an exhaustive epidemiologic study of all patterns of glioma spread in cases around the Sylvian fissure. It is possible that a different center may find different frequencies of network involvement with a similar cohort. However, the fact remains that the rate of these networks being involved in these tumors is likely high. The initial clinical experience of these cases we reviewed may not be able to represent the complexity of insulo-Sylvian gliomas, and we have not got their long-term functional status in the follow-up. Although our study aimed to point out the possible way to decrease the risk of cognitive morbidity in the majority of patients and provide insights into the potential of Quicktome in surgical planning from preoperative discussions to intraoperative decision-making, we recognized that the application of Quicktome will have to be explored systematically to ensure external generalizability.

## Conclusions

In this study, we demonstrate the extensive involvement of insulo-Sylvian tumors with non-traditionally “eloquent” brain networks involved in cognition. Brain network maps provide an improved understanding of the relationship between tumor and brain anatomy such that neurosurgeons can better understand the risks and benefits of certain surgical decisions for specific tumors. With the assistance of Quicktome, we can optimize the surgical approach and the appropriate border of surgical resection, to preserve the important brain networks and improve the patient’s functional status after surgery and thus maintain their quality of life.

## Data Availability

All identified data used in the current manuscript can be provided by the authors per the readers’ requests. No data spreadsheet has been made publicly available due to patient confidentiality.

## Abbreviations

SN: Salience network
CEN: Central executive network
DMN: Default mode network
DAN: Dorsal attention network
VAN: Ventral attention network
HCP: Human Connectome Project
DTI: Diffusion tractography imaging
MMSE: Mini-Mental State Examination
FDA: Food and Drug Administration
MoCA: Montreal Cognitive Assessment
BOLD: Blood oxygen level dependence
